# Brain mechanisms underlying apathy

**DOI:** 10.1136/jnnp-2018-318265

**Published:** 2018-10-26

**Authors:** Campbell Le Heron, Clay B Holroyd, John Salamone, Masud Husain

**Affiliations:** 1 Nuffield Department of Clinical Neurosciences, University of Oxford, Oxford, UK; 2 Department of Experimental Psychology, University of Oxford, Oxford, UK; 3 New Zealand Brain Research Institute, Christchurch, New Zealand; 4 Department of Psychology, University of Victoria, Victoria, British Columbia, Canada; 5 Department of Psychological Sciences, University of Connecticut, Storrs, Connecticut, USA; 6 Division of Clinical Neurology, John Radcliffe Hospital, Oxford University Hospitals Trust, Oxford, UK; 7 Wellcome Trust Centre for Integrative Neuroimaging, Oxford, UK

**Keywords:** apathy, motivation, goal-directed behaviour, reward, effort, decision making, cognitive neuroscience

## Abstract

The past few decades have seen growing interest in the neuropsychiatric syndrome of apathy, conceptualised as a loss of motivation manifesting as a reduction of goal-directed behaviour. Apathy occurs frequently, and with substantial impact on quality of life, in a broad range of neurological and psychiatric conditions. Apathy is also consistently associated with neuroimaging changes in specific medial frontal cortex and subcortical structures, suggesting that disruption of a common systems-level mechanism may underlie its development, irrespective of the condition that causes it. In parallel with this growing recognition of the clinical importance of apathy, significant advances have been made in understanding normal motivated behaviour in humans and animals. These developments have occurred at several different conceptual levels, from work linking neural structures and neuromodulatory systems to specific aspects of motivated behaviour, to higher order computational models that aim to unite these findings within frameworks for normal goal-directed behaviour. In this review we develop a conceptual framework for understanding pathological apathy based on this current understanding of normal motivated behaviour. We first introduce prominent theories of motivated behaviour—which often involves sequences of actions towards a goal that needs to be maintained across time. Next, we outline the behavioural effects of disrupting these processes in animal models, highlighting the specific effects of these manipulations on different components of motivated behaviour. Finally, we relate these findings to clinical apathy, demonstrating the homologies between this basic neuroscience work and emerging behavioural and physiological evidence from patient studies of this syndrome.

## Introduction

The importance of outcomes for motivating behaviour has long been recognised. Philosopher and physician John Locke ascribed a crucial role for reinforcers (*pleasure* and *pain*) for motivating actions, writing that without these perceptions

*… we should have no reason to prefer one thought or action to another … and so we should neither stir our bodies, nor employ our minds, but let our thoughts (if I may so call it) run adrift … In which state man … would be a very idle, inactive creature, and pass his time only in a lazy lethargic dream.* (s1)

This description resonates with much of the phenotype of apathy, a neuropsychiatric syndrome conceptualised as a loss of motivation that manifests in reduced goal-directed behaviour^1 2^—see box 1 for key definitions. Other terms often used to describe this syndrome include abulia, akinetic mutism, athymhormia and autoactivation deficit (see online supplementary table 1 for proposed diagnostic criteria). Apathetic patients often report that they “*can’t be bothered,*” that activities “*don’t seem worth it,*” or that they “*just don’t know*” why they no longer engage in behaviours they used to do (box 2). However a mechanistic understanding of this syndrome remains elusive.

10.1136/jnnp-2018-318265.supp1 Supplementary data

Box 1 **Key terms and abbreviations**

- *Apathy*: a syndrome of impaired motivation and consequent reduced goal-directed behaviour.
- *﻿* *Motivation:* a construct that encompasses the reasons and processes underlying behaviour directed towards or away from environmental stimuli.
- *Goal-directed behaviour:* actions towards an outcome, in which the current outcome value for the organisms, as well as the costs associated with the action, are accounted for.
- *Habitual behaviour:* actions that occur in response to a particular stimulus, without explicit reference to the outcome or costs of the action. Such ‘stimulus-response’ systems are shaped by learning systems which compute whether an action results in a better or worse than predicted outcome, making the same action more or less likely to be repeated on subsequent exposure to the stimulus.
- *Reward:* this term has a clear meaning when used as a noun (ie, food reward) or a verb referring to actions (to reward someone). However, when used as a neurobehavioural process, this term has no standard usage and can be quite ambiguous. In some contexts, it is used as a synonym for pleasure. In others, it is used as a synonym for reinforcement. Thus, overuse of the term reward can be confusing. In this paper, when used, reward refers to a reinforcer.
- *Reinforcer:* the positive (reward) or negative (punishment) outcome of an action that informs goal-directed behaviour and also shapes subsequent habitual behaviours.
- *Effort:* physical or mental work or activity done to achieve a particular end or goal; vigorous exertion of power.
- *Instrumental:* the phase of motivated behaviour during which an organism is approaching a motivational stimulus or behaving so as to deliver that stimulus or increase its probability of occurrence; also referred to as appetitive, or ‘seeking’ behaviour.
- *Consummatory:* the phase of motivated behaviour during which an organism directly interacts with the motivational stimulus (eg, intake of food or water); also referred to as ‘taking’.
- *Effort-based decision making* *(* *EBDM* *)*: this can refer to specific behavioural tasks in which an organism must choose between a preferred reinforcer that requires high exertion of effort and a less preferred reinforcer requiring no or little effort (also termed effort-based choice). EBDM also refers to a more generalised framework centred around exertion of effort for reinforcers, including effort-based choice, vigour and persistence towards goals and learning about the outcomes of actions.
- *Vigour:* the intensity or local rate of an instrumental behaviour.
- *Persistence:* the maintenance of behaviour over time. Motivated behaviour is often characterised as being vigorous and persistent.
- *Prediction* *e* *rror:* the discrepancy between the outcome value and the earlier prediction of what this value would be. Strong evidence supports the view this is encoded by fast (phasic) changes in dopamine levels and drives learning processes to influence future behaviour.

Box 2 **Transcript of an interview with a patient with significant apathy in the context of cerebral small vessel disease**

Doctor: *“If I was a fly on the wall watching you at home, what would I see you doing?”*

Patient: “I suppose sitting, watching television which drives him mad, … (pauses) …, I did use to book-read but I don’t seem to be doing that much these days. Um, and that’s basically it, really.”

Doctor: *“So watching the television?”* (Patient nods). *“Anything else?”*

Patient: “I can’t think of anything, really.”

Doctor: *“In the past, would that have been you?”*

Patient: “No”.

Doctor: *“What would we have seen if we had been a fly on the wall a few years back?”*

Patient: “Me buzzing around, doing things, going out shopping, coming back, you know, being busy all the time. And I’ve just slowed down and more or less come to a halt. I just don’t know what is stopping me really.”

Doctor: *“You said you used to enjoy reading. So what’s stopping you reading?”*

Patient: “Absolutely nothing. It’s just that I can’t be bothered I suppose. It’s easier to watch a screen than read a book. I keep threatening to pick up a book but I just don’t get there.”

Doctor: *“What else did you use to do in the past? You said you used to run the house?”*

Patient: “I used to sew. I used to sew all my daughter’s clothing. Knit. But, it seems to all have gone … I don’t do any of that now and I don’t know why.”

Doctor: *“Would you like to?”*

Patient: (pauses) … “Umm. Part of me wants to say yes, and part of me says I can’t be bothered.”

Doctor: *“How would you describe your mood?”*

Patient: “Well I think that my mood is good and always is.”

Doctor: *“What about things that give you pleasure? Are there things that make you laugh?”*

Patient: (laughing) “Annoying him!” (husband); “sorry” (still laughing).

Doctor: (laughing) *“Apart from that? Are there any other things that give you pleasure, that you like doing?”*

Patient: “My daughter often takes me out to the cinema which I enjoy very much. Umm, I really don’t do much else.”

Although prevalence estimates vary widely depending on assessment tools and the clinical population studied, there is no doubt that apathy is a common accompaniment to a broad range of brain disorders (table 1). A number of neurodegenerative, vascular, inflammatory, infectious and traumatic brain pathologies have been associated with the development of apathy, while it is also a component of the negative symptoms of schizophrenia and anhedonia associated with major depressive disorder. It can be the first clinical marker of a developing condition, as in Parkinson’s disease (PD), and in some conditions (eg, Huntington’s disease) apathy has been shown to closely track disease progression (s2). Importantly, its presence is associated with worsened quality of life for both patients and their families/caregivers, highlighting the clinical significance of this syndrome.^3 4^ Indeed, the presence of apathy may negate the otherwise positive effects of interventions targeting other aspects of disease, such as deep brain stimulation for the motor symptoms of PD.^5^

**Table 1:** Apathy occurs commonly ﻿in a broad range ﻿of neurological and psychiatric conditions

Condition	Prevalence	Comments
Parkinson’s disease (PD)	17%–70% (likely ~30% in the general population)	A common non-motor symptom of PD. Occurs at all stages of the disease and is a presenting symptom in >20% of patients. Clear evidence it is an intrinsic (rather than reactive) feature of PD.^3 50^
Alzheimer’s disease	~50%	Along with PD, apathy in AD probably has had the greatest level of research interest to date, including epidemiology, imaging correlates and therapeutics (s16).
Sporadic cerebral small vessel disease (SVD)	15%–30%	An increasingly recognised complication of SVD, with a clear association between apathy and both background vascular risk factors and imaging changes of SVD (s17, s18).
CADASIL	40%	One of the cardinal features of this condition. Occurs at all clinical stages but more likely with progression of the disease (s19).
Stroke (large vessel territory)	~30%	Occurrence is associated with worse outcomes and poorer quality of life^4^ (s20).
Frontotemporal dementia (FTD)	>50% (particularly behavioural variant)	A core feature of behavioural variant FTD (which can have different underlying pathologies), although also present in other subtypes. Strongly associated with impulsivity (s21).
Progressive supranuclear palsy (PSP)	Up to 90%	Occurs in nearly all patients with PSP (s22).
Corticobasal syndrome	50%–90%	A common feature of this neurodegenerative syndrome, which is associated with different underlying pathologies (s23).
Amyotrophic lateral sclerosis	40%	A common feature, with at least mild apathy symptoms present in most patients (s24).
Huntington’s disease	>30%	Common and strongly related to disease progression (s2).
Traumatic brain injury	20%–72%	Increasingly recognised as a sequalae (s25).
HIV infection	25%–40%	A common sequelae of HIV infection, it has been related to the extent of brain pathology particularly within the ventral striatum and the subcortical white matter (s26, s27).
Multiple sclerosis (MS)	22%	Emerging evidence suggests apathy is a common neurobehavioural feature of MS (s28).
Myotonic dystrophy	40%	A noted feature of this condition, although limited research to date (s29).
Wilson’s disease	24%	Reported in some patients in a single study (s30).
Depression	38%	Dissociable from, but associated with the syndrome of depression, particularly anhedonic components (s31).
Schizophrenia	47%	One of the core components of the negative symptoms of schizophrenia (s32).

References are provided in the online supplementary material.

CADASIL, Cerebral autosomal dominant arteriopathy with subcortical Iinfarcts and leukcoencephalopathy.

10.1136/jnnp-2018-318265.supp2 Supplementary data

Since the work of both Marin^1^ and Levy and Dubois,^2^ apathy has generally been considered as divisible into three core components, each of which could contribute to (or be the manifestation of) reduced goal-directed behaviour/motivation. These are an *affective/emotional* component, a *behavioural activation* component and a *cognitive* component. Furthermore, Levy and Dubois^2^ proposed that these components may relate to disruption of distinct frontal cortex-basal ganglia circuits. Many questionnaires used to assess apathy include subscales that purport to map onto these separate components.^6^ This approach has undoubtedly been important in advancing understanding of the apathetic syndrome. However it remains unclear how these components map onto underlying normal neurobiological systems, the disruption of which are the presumed underlying drivers of apathy in the first place.^7^ Furthermore these components have not been extensively studied in humans or animal models. Therefore in this review we prefer to develop a framework for apathy based on current understanding of the processes underlying normal motivated behaviour—processes we review in more detail below.

Studies examining the neuroimaging correlates of the apathetic syndrome have identified consistent associations between apathy and disruption of specific medial frontal cortex and subcortical structures—see refs^7 8^ for reviews. These include anterior cingulate cortex (ACC), medial orbitofrontal cortex (OFC) and ventral striatum (VS). Strikingly, these changes are present across a disparate range of underlying pathologies and identifiable using multiple imaging techniques. This suggests a crucial role for these structures in the development of apathy, and the possibility that it is the disruption of specific brain systems and cognitive processes—rather than any specific molecular pathology causing this disruption—that matters most in its aetiology.

In parallel with the growing recognition of the clinical importance of apathy, significant advances have been made in our understanding of normal motivated behaviour in humans and animals. These advances have occurred at several conceptual levels, from research that links particular neural structures and neuromodulatory systems to specific aspects of motivated behaviour,^9–12^ through to higher order computational models of neural function that aim to unite these findings within frameworks that explain the production of normal goal-directed behaviour.^13^ With these advances comes an opportunity to understand complex neuropsychiatric syndromes, such as apathy, in terms of dysfunction of normal underlying cognitive processes.

## The neuroscience of motivated behaviour

**Figure 1:**
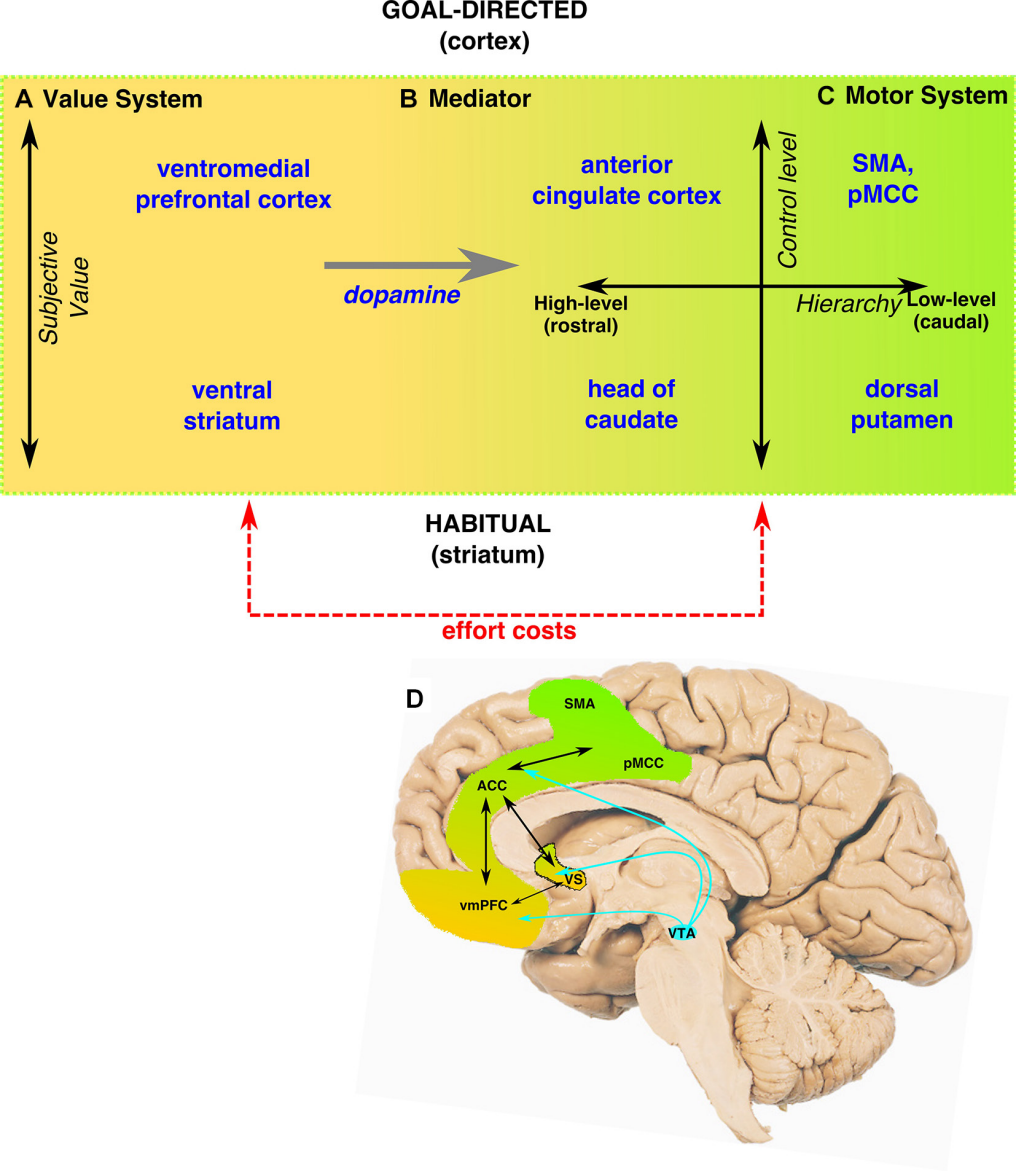
Conceptual framework and brain basis of motivated behaviour. The translation of value (reinforcer) information into a behavioural response can be conceptualised as three distinct processes. (A) A valuation system computes the subjective value (ie, allowing for current internal states and costs to obtain the reinforcer) of the current and potential events. (B) A mediating system integrates this reinforcer/cost information to activate the motor system towards particular goals. (C) A motor system produces behaviour towards motivationally relevant stimuli. Furthermore, these processes occur along two distinct neurocognitive dimensions (right side of panel). (1—vertical axis) Behaviour can be activated by flexible, but computationally demanding, goal-directed systems, which actively represent the outcome of potential actions along with the costs of these actions. It can also be activated by inflexible, simpler habitual systems, which activate responses based on previously learnt stimulus-response mappings. (2—horizontal axis) Motor control functions are hierarchically organised, such that complex behaviours are represented as higher level, abstract actions (eg, fly to London) as well as the lower level subcomponent behaviours (eg, book a flight, move mouse cursor upwards…). Outcome feedback and learning occur at all hierarchical levels to inform future behaviour. Such hierarchical arrangements likely occur within both goal-directed (predominantly cortical) and habitual (predominantly subcortical) systems. (D) These processes are instantiated within a complex network of reciprocally connected cortical and subcortical brain regions, under the influence of the mesolimbic dopaminergic system. A single brain region likely contributes to more than one process, but with specialisation. Hence the value system predominantly involves the vmPFC and, the motor system predominantly pMCC, SMA, ACC and the dorsal striatum (including the caudate and the putamen), while the VS, ACC and mesolimbic dopamine (originating in VTA) form the mediating system. This gradient of function is represented in the figure by the gradual change from gold (reinforcer) to green (motor). ACC, anterior cingulate cortex; pMCC, posterior mid-cingulate cortex; SMA, supplementary motor area; vmPFC, ventromedial prefrontal cortex; VS, ventral striatum; VTA, ventral tegmental area.

Motivated behaviour in humans and other animals is characterised by active efforts to obtain positive reinforcers (rewards). This truism suggests three fundamental processes at work. First, an internal valuation system must determine the subjective value of ongoing events in terms of their hedonic or aversive potential, as well as their potential costs, including energy expenditure (effort) and temporal proximity (figure 1A). Second, a motor system must act on the environment in order to pursue outcomes with high value and to avoid aversive events (figure 1C). And third, a mediating system, under the influence of the value system, must activate the motor system towards particular goals (figure 1B).

The *value system* includes the VS (comprising the nucleus accumbens (NAc) and adjacent areas in the rostral caudate nucleus and the ventral-rostral putamen) and the ventromedial prefrontal cortex (vmPFC), which includes the most rostral areas of ACC and adjacent areas of medial OFC^14^ (figure 1A). Although VS and vmPFC are reciprocally interconnected, they may differ in the information they encode. There is evidence that the VS mediates a process of learning, via conditioning, which states of the environment predict future rewards.^15^ For example, a cigarette smoker might learn by such a process that the sight of a package of cigarettes predicts the reward value of a forthcoming smoke. Because these associations are developed through a gradual process of reinforcement, they can be difficult to dispel. By contrast, the hallmark of vmPFC function is its flexibility: although activity within vmPFC is also sensitive to the subjective value of events,^16^ there is evidence that this activity varies dynamically as if such values are being reassessed on the fly.^14^ Neural activity in vmPFC also parametrically varies with the subjective value of multiple different reward types, which allows for comparison across qualitatively different types of outcomes and reinforcers on a common scale.^17^ This flexibility appears to support rapid shifts in preference, for example, as when a dieter favours high-protein foods one day and liquid foods the next.

Once computed, this value information must be translated into appropriate behavioural responses (figure 1B). The neural circuitry underlying this *mediating system* is complex and distributed. Although some theories have proposed a hard distinction between valuation and motor systems,^15^ it is widely accepted in behavioural neuroscience that both VS and ACC serve as crucial ‘limbic/motor’ interfaces, under the influence of the mesolimbic dopamine (DA) system, which originates in the ventral tegmental area of the midbrain and projects widely to these regions and related structures^18^ (figure 1D). Moreover, although DA is a key modulator, other neurotransmitter systems may also play a crucial role.^19 20^

The DA system carries at least two reward-related signals (but see Berke^21^). Fast or phasic (100–200 ms) increases and decreases in DA neuron firing-rate convey the so-called reward prediction error signals that indicate when ongoing events are ‘better’ or ‘worse’ than expected. These signals enable the neural targets of the DA system to learn what actions and events elicit positive reinforcers, which can in turn guide future behaviour.^22^ Additionally, slower or tonic DA signals are thought to regulate levels of physical and cognitive activation, including vigour of response, towards goals^11 21 23^ (s3). Finally, the production of behaviour is mediated by the *motor system*, including posterior mid-cingulate cortex, supplementary motor area and dorsal striatum, under the influence of these inputs (figure 1C). We emphasise that, although conceptually useful for understanding the different components contributing to normal motivated behaviour, it is unlikely that the proposed valuation, mediating and motor systems exist as discrete entities within the brain. Rather, these processes are likely to be instantiated neurally along anatomical and functional gradients (s4) (figure 1—background colour scheme﻿).

## Dimensions of motivated behaviour

Over the past few decades, research in artificial intelligence has yielded several computational algorithms that realise these principles in different ways (s5). Drawing on these insights, parallel work in cognitive neuroscience has suggested that these processes express in living agents along two key dimensions of neurocognitive function (figure 1). First, the brain appears to distinguish between inflexible, habitual (so-called model-free) behaviours, which according to some accounts are processed mainly by the striatum; and flexible, goal-directed (so-called model-based) behaviours, which are processed mainly by the frontal cortex (vertical dimension in figure 1﻿).^24^ Second, motor control functions appear to be represented hierarchically along the medial frontal cortex, with more rostral areas responsible for the most complex aspects of motivated behaviour (horizontal dimension in figure 1C).^25^

Early studies of habitual behaviour focused on the motor functions of the basal ganglia, especially within the dorsal striatum (figure 1C, bottom half), where DA reward prediction error signals were hypothesised to reinforce rewarding actions.^15^ Comparable with the imprinting of reward predictions instantiated within the VS (figure 1A), this process is thought to solidify actions into habits that are easy to execute but inflexible and difficult to overcome. In contrast, a goal-directed system associated with the prefrontal cortex is thought to use an internal model of the environment (encompassing the current (subjective) value of potential reinforcers and the effort costs required to obtain them) to flexibly adapt behaviour (figure 1C, top half).

This flexibility is considered to be especially useful when planning sequences of goal-directed actions.^24^ But its power comes at a cost: goal-directed actions demand greater brain resources than do habitual actions (s6). Together, this dual-systems mechanism allows a *goal-directed* approach to novel problems (like figuring out what to eat when starting a new diet), with control subsequently moving to the *habitual* system once the problem has been overlearnt (like automatically reaching for a healthy snack after the diet has been internalised), thereby freeing resources within the prefrontal cortex to be directed to the next problem.

Yet despite their complementary strengths, as described so far, both goal-directed and habitual systems would struggle with real-world problems that demand extended chains of actions. For example, a holiday to a foreign country might stymie both the habitual system (because every action on the trip would require repeated reinforcement, which is an unlikely prospect even for consummate globetrotters) and the goal-directed system (because, due to capacity limits, planning would be overwhelmed by a combinatorial explosion of potential action sequences).

This computational challenge has led to a possible solution: that individuals reframe complex action sequences hierarchically. Rather than making a series of choices between low-level actions (like turning on the computer, opening a browser, searching for a travel website and so on), the system instead selects between relatively abstract, high-level actions (like buying a ticket, taking a taxi to the airport, going to the gate and so on). Then, once a high-level action has been selected and put into execution, the details of the action fall to lower level systems for implementation. It has recently been proposed the rostral-caudal extent of the medial frontal cortex contributes to such hierarchical encoding^23 25^ (horizontal axis of figure 1C﻿).

At the apex of both goal-directed and hierarchical systems lies the ACC.^23 26 27^ This conjunction of hierarchically organised action selection with model-based planning enables the development and execution of plans that lower level systems might have trouble implementing. Imagine planning a trip to a foreign country to celebrate an important anniversary only to find that your ride to the airport has been delayed, necessitating a luggage-encumbered sprint to the gate. Success might require that the higher level system enforce its plan, especially should the lower level systems—which are oblivious to the personal importance of the trip for you—baulk at its demands. Thus, it has been proposed that a critical feature of ACC function may be *to marshal the effort necessary to execute hierarchically organised, goal-directed behaviours*.^23 26^ This process is likely supported by DA signals and reciprocal connections with the VS and vmPFC that enable learning about both the subjective value as well as the effort costs of actions.^12 23^ As we discuss below, weighing up the subjective value of a potential course of action against the effort required is an important theoretical framework for understanding motivation and apathy.

## Deficits in behavioural activation in animals

**Figure 2:**
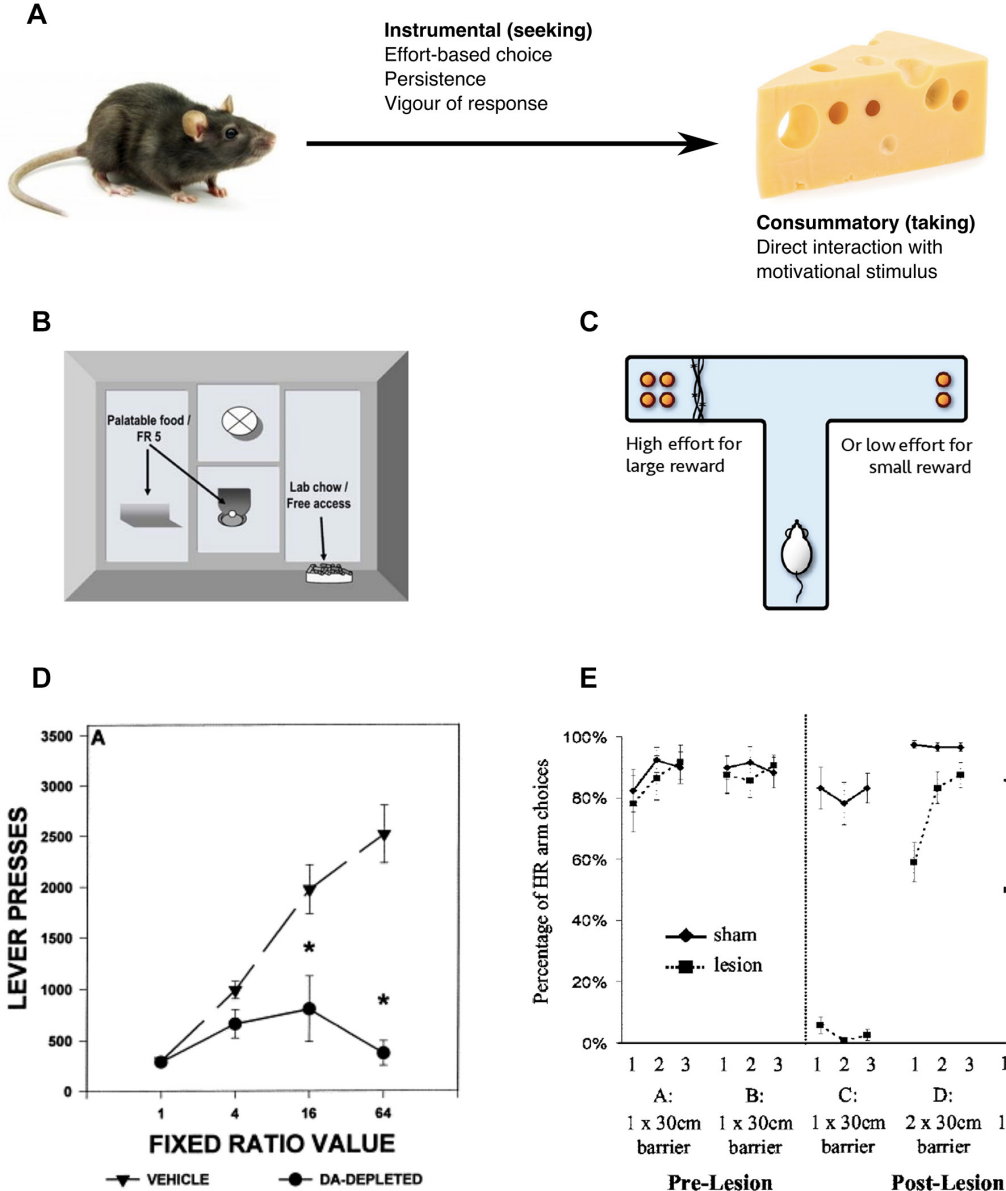
Effects of dopaminergic and anatomical lesions on phases of motivated behaviour. (A) Motivated behaviour takes place in two distinct phases. The *consummatory* phase involves direct interaction with the motivational stimulus, whereas the *instrumental* phase describes the behaviours required to obtain this stimulus. Instrumental behaviours are both *directional* (towards or away from stimuli) and *activational* (energising), allowing organisms to overcome obstacles separating them from salient stimuli. Thus, organisms pay response ‘costs’ to access motivationally relevant goals. (B) Effort-based choice is often assessed in rodents using one of two experimental set-ups. Animals can be free to choose between pressing a lever (effort cost) a fixed number of times (ratio can be varied) to receive a preferred reinforcer, or accessing standard lab chow (less preferred reinforcer) at any point. (C) Alternatively a T-maze set-up allows animals to choose between a high-reward, high-effort cost option and a low-reward, lower cost option. (D) Both low-dose systemic dopamine antagonists and local (nucleus accumbens) dopamine depletion or antagonism bias responses towards low-effort, low-reward options, without affecting reward preference (when effort costs are equalised) or motor ability. Thus interference with the dopaminergic system reduces animals’ willingness to undertake costs to access motivationally relevant goals. (E) Anterior cingulate cortex lesions produce very similar changes in behaviour. Prior to the lesion, rodents preferred to climb a 30 cm barrier to obtain high rewards (A and B on graph). Post lesion, the behaviour changed dramatically, with rodents now choosing the low-effort, low-reward option (C). However, equalising effort costs led to rodents again choosing the high-reward option (D), indicating reward preference was not altered by the lesion. Adapted from Aberman and Salamone, 1999 (s33); Walton *et al* 10 with permission. DA, dopamine; HR, high reward (arm)

Because motivationally relevant stimuli are typically at some psychological or physical distance away from organisms, motivated behaviour takes place in phases.^28^ While the direct interaction with a motivational stimulus is typically referred to as the *consummatory phase* (also known as *‘* *taking* *’*), the behaviours that must be engaged in to gain access to the motivational stimulus are generally referred to as *instrumental* in nature (or *‘* *seeking* *’*) (figure 2A **﻿**). Central to the construct of motivation is that instrumental behaviours have clear *directional* aspects, being driven towards or away from particular stimuli or conditions—that is, they are ‘goal directed’. However, another fundamental feature of motivation is its *activational* aspects. Motivated behaviour is often characterised by high levels of activity, vigour and/or persistence,^11 28^ factors that are often considered as part of an ‘energizing’ component of motivation.^29^

These activational or energising aspects of motivation can be highly adaptive because they enable organisms to overcome obstacles that separate them from conditions that are critical for survival. Thus, in terms that make reference to concepts in the field of behavioural economics, organisms must pay the work-related response costs that allow access to motivationally relevant goals. This can be seen in animals foraging in the wild, as well as animals running in mazes or pressing levers in laboratory experiments. Moreover, these features of motivation are evident in humans striving vigorously for a career or life goal. Over the last few decades, animal research has greatly expanded our understanding of the role played by the mesolimbic DA system, along with the neural regions including the VS and the ACC, in effort-related aspects of motivation, including the exertion of effort during the performance of instrumental behaviour tasks, and effort-based decision making (EBDM).^11 28^

### Animal studies of EBDM

Studies of *effort-based decision making* traditionally offer organisms a choice between access to a preferred reinforcer that can be obtained only via a high-effort instrumental action versus a less preferred but low-effort option (see ref 11 for review). One commonly used procedure involves giving an animal a choice between operant lever pressing to receive a preferred reinforcer (eg, high-carbohydrate pellets) versus approach and consumption of a less preferred reinforcer (eg, standard lab chow) that is freely available in the chamber (s7) (figure 2B). Typically a fixed ratio (eg, five presses) or a progressive ratio (increasing number of presses each trial) paradigm is used. Alternatively, in T-maze effort-based choice procedures, one arm of the maze contains a higher density of food reward that can be obtained only by climbing a barrier, whereas the alternative choice is to access an arm that contains less food, but has no or a lower barrier^10 30^ (figure 2C).

The preponderance of research on such effort-based choice has focused on the role of DA systems. DA antagonists with varying degrees of D1 or D2 family selectivity, whether administered systemically or directly into the NAc, consistently produce shifts in choice behaviour marked by decreased selection of the high-effort option and a bias towards selecting the low-effort option^28^ (figure 2D). This low-effort bias is also induced by neurotoxic or pharmacological depletion of NAc DA.^19^ In contrast, medial striatal DA antagonism or depletion generally fails to induce alterations in effort-based choice,^19^ and lateral striatal DA depletions produce severe motor coordination impairments that non-specifically affect performance (s8). Therefore, changes in effort-based choice associated with DA manipulations are specific to NAc manipulations.

Importantly, a number of control experiments have clearly demonstrated that these effects cannot be explained by other factors. In particular, a strong literature demonstrates that DA depletion does not alter reinforcer preference when effort costs to obtain the reward are equalised, meaning there is no evidence that the effects of impaired DA transmission on effort-based choice are mediated by some kind of ‘reward’ or ‘liking’ impairment.^19 31^ Similarly, these effects cannot be attributed to deficits in motor incapacity. For example, in studies using the T-maze barrier task, it has been shown that DA depletions that induce a low-effort bias do not impair the ability of animals to climb the barrier if that is the only way to obtain the food (s9).

In summary, low doses of DA antagonists or NAc DA antagonism or depletion affects behavioural activation functions that represent areas of functional overlap between motivational and motor function, crucially without producing broad deficits in either reward preference (when effort costs are equal) or motor incapacity. In terms of behavioural economics concepts, interference with DA transmission effectively dissociates ‘willingness to pay’ from preference or utility, which could reflect a reduced perception of behavioural resources available for paying effort-based costs.^11^ That is, DA depletion reduces willingness to undertake effort costs in order to access motivationally relevant goals.

Of course, no one transmitter or brain area mediates a behavioural function in isolation. Mesolimbic DA is part of a distributed forebrain circuitry that includes the basolateral amygdala, prefrontal/anterior cingulate cortex and ventral pallidum, and anatomical lesions of these sites produce strikingly similar changes in choice preference (towards low-effort, low-reward options) as DA manipulations^10 32^ (s10) (figure 2E). Furthermore, a number of transmitters and neuromodulators in addition to DA participate in the regulation of effort-related choice, including acetylcholine, adenosine, serotonin and gamma-aminobutyric acid (GABA).^19 20^ The distributed nature of this circuitry offers challenges to researchers attempting to establish the contributions of each component, but also offers opportunities for exploring potential pharmacological strategies that can be developed for clinical intervention (see online supplementary material—Animal models of motivational dysfunction).

## Apathy in the context of motivated behaviour

**Figure 3:**
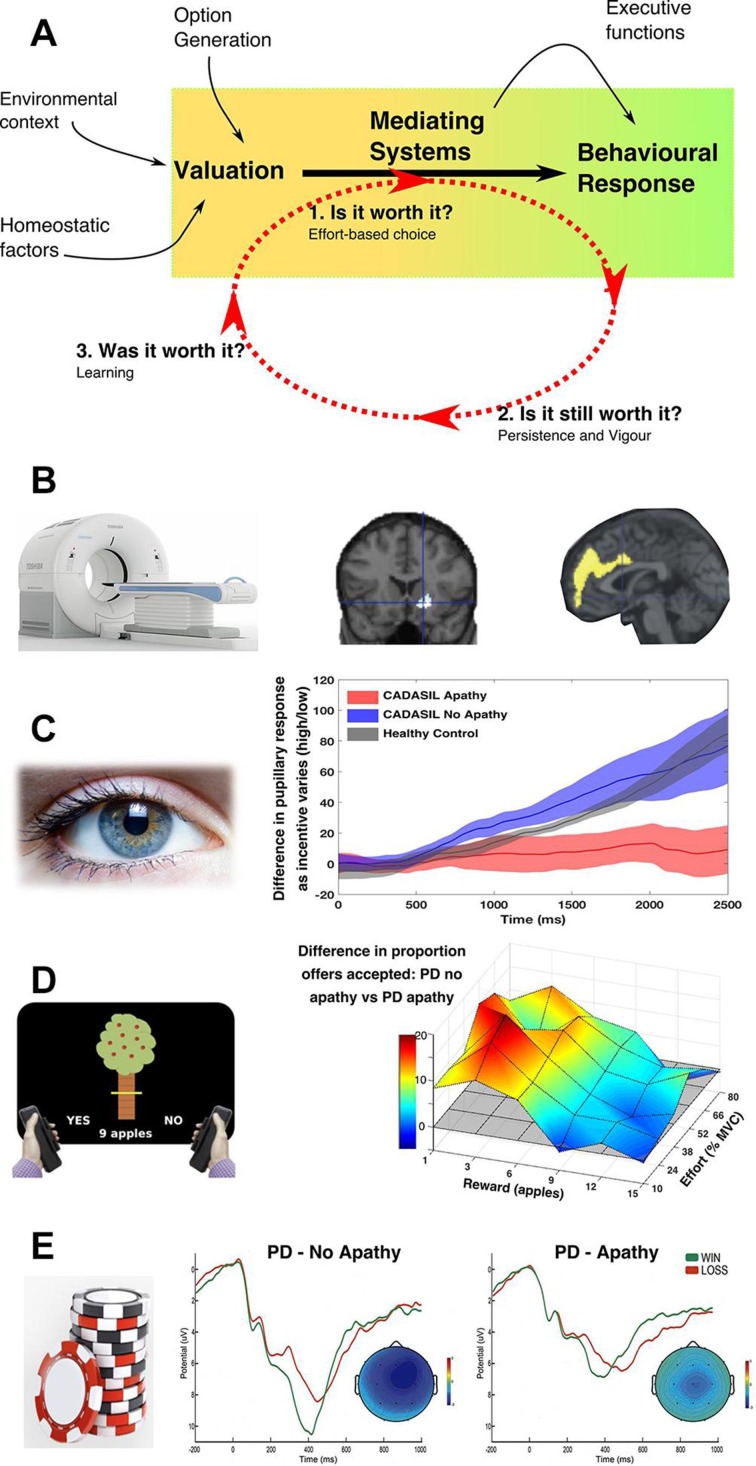
A neurocognitive framework for apathy. (A) Apathy could result from alterations in the processes underlying the translation of value information to actions, which are often described under the term *effort-based decision making*. Reduced willingness to exert effort for reward (effort-based choice—’*is it worth it’*), as well as impaired persistence and vigour towards goals (‘*is it still worth it’*), could lead to the reduced goal-directed behaviour that characterises apathy. Similarly changes in learning from the reinforcing outcomes of actions could change the likelihood of the same behaviour being repeated in the future (’*was it worth it’*). These changes could be driven by anatomical disruption of key neural regions (such as the anterior cingulate cortex and ventral striatum) and/or changes in neuromodulators such as the mesolimbic dopaminergic system. (B) A large number of studies, across disorders and using different imaging modalities (eg, positron emission tomography), have associated disruption of the ventral striatum and anterior cingulate with apathy. (C) Reward sensitivity can be assessed by measuring the degree of pupillary dilatation to reinforcer information. Apathy is associated with impaired autonomic (pupillary) responses to reward in both genetic cerebral small vessel disease (shown) and PD. (D) Effort-based choice can be assessed by sequentially offering patients varying levels of reward in return for exerting varying levels of effort. Apathy is associated with reduced willingness to exert effort for reward. This change is not global, but is driven by reduced sensitivity to rewarding outcomes without a change in sensitivity to effort costs, in both patients with genetic small vessel disease and PD (shown). (E) Gambling tasks, in which patients select an option and are then provided feedback about whether they ‘won’ or ‘lost’, allow assessment of outcome-related physiological changes, which are thought to drive learning. PD patients with apathy show blunted outcome-related electrophysiological activity compared with non-apathetic patients. Adapted from Le Heron *et al* 2018^35 38^, Martínez-Horta *et al,* 33 Robert *et al* 2014^34^ and Schroeter *et al* 2013^35^ with permission. CADASIL, cerebral autosomal dominant arteriopathy with subcortical infarcts and leucoencephalopathy; MVC, maximal voluntary contraction; PD, Parkinson’s disease.

The body of work reviewed above, spanning basic neuroscience research in animal models to computational and imaging methods in healthy humans, emphasises a distributed but interconnected network of brain regions operating to produce motivated behaviour, under the influence of neuromodulators such as DA. Furthermore, structural or pharmacological disruption of this network in animal models reduces the effort that animals are prepared to exert to obtain rewards, their willingness to engage with goal-directed activities, their persistence and vigour of actions towards these goals, and their ability to learn from outcomes.^11^ Although caution should always be exercised in extrapolating the results of animal studies to human behaviour, these features have a striking similarity to aspects of the clinical syndrome of apathy. Moreover, the very regions identified in studies of normal motivated behaviour—in particular ACC and VS—have been implicated in clinical apathy across several different disorders and imaging modalities^7^ (figure 3B).

### EBDM in apathy

As discussed above, the cognitive processes involved in the activation and maintenance of behaviour—or sequences of behaviours—towards goals are often described as elements of EBDM. They include choosing whether to perform actions towards a goal, sustaining behaviour with reference to background reward context and learning through monitoring outcomes of a behaviour. In other words: *Is it worth it, is it still worth it* and *was it worth it*? EBDM thus provides a neurobiologically grounded framework within which to investigate the cognitive mechanisms that may underlie apathy, a framework on which empirical studies are beginning to build (figure 3).

#### Integration of rewards and effort costs to drive goal-directed behaviour

As noted previously, the process of integrating reward (reinforcer) and effort information to drive behaviour may occur simultaneously at multiple hierarchical levels (individual action, subgoal, goal) and is strongly influenced by the mesolimbic dopaminergic system. An important question is whether apathetic patients fail to undertake goal-directed activities because they are *insensitive* to the rewards associated with the activity, or whether they are instead *hypersensitive* to effort costs. Either change could reduce an integrated value signal, making activation of behaviour towards a goal less likely to occur.

There is emerging physiological evidence that reward processing is altered in apathetic patients, such that they are insensitive to rewarding outcomes.^33 34^ Pupillary dilatation to incentives provides an autonomic marker of motivation (s11). Blunting of this normal pupillary response has now been demonstrated in two separate, clinically apathetic patient groups—PD and cerebral autosomal dominant arteriopathy with subcortical infarcts and leucoencephalopathy (CADASIL)—as they performed a saccadic eye movement task for rewards^34 35^ (figure 3C). Similarly, reduced activation of neural regions including the VS and OFC in response to reward information during functional MRI (fMRI) studies has been associated with amotivation in patients with schizophrenia.^36 37^

These physiological alterations in reward processing are mirrored by behavioural changes. In separate studies in which patients decided whether or not to exert physical effort for rewards, both apathetic patients with PD and CADASIL were less willing to accept offers. Crucially, this change in response was not global across the sampled options, but instead was driven by *reduced sensitivity to rewards, but not increased sensitivity to effort costs*. In other words, apathetic patients were prepared to exert even high levels of effort, but only if rewards were high enough^35 38^ (figure 3D). Furthermore, this reduction in reward incentivisation was associated with greater blunting of pupillary responses to reward, linking physiological evidence of reduced reward sensitivity in apathy to a behavioural consequence.

Changes i^35^ n effort-based choice associated with apathy have also been demonstrated in patients with schizophrenia,^37 39^ and related to levels of motivation and DA state in non-apathetic patients with PD.^40^ These results are complemented by an fMRI study in which changes in the blood oxygen level dependent (BOLD) signal were measured as healthy participants performed a similar decision-making task. Higher levels of apathy traits in this group were associated with individual differences within the ACC and related medial frontal regions considered to be crucial for marshalling the effort to execute such goal-directed behaviours^23 41^ (figure 1).

Intriguingly, in PD the effect of apathy on choice was dissociable from the effect of DA depletion (via an overnight withdrawal of patients’ normal dopaminergic medications). In contrast to apathy’s effect on *low-reward* options, depleting DA reduced acceptance of options which required *high effort for high rewards*,^38^ *﻿* consistent with animal models of DA depletion.^11^ Together, this work suggests that measures to increase the incentivising value of rewards are an important therapeutic target, but simply increasing dopaminergic tone—while increasing activation towards high reward options—may not specifically alter the underlying apathetic state.^38^

#### Persistence

Persisting with an action-set towards a temporally or spatially remote reward is a crucial aspect of instrumental behaviour.^28^ Furthermore, like other components of instrumental behaviour such as the vigour of response, persistence is strongly influenced by the mesolimbic dopaminergic system, and particularly its projections to VS and ACC.^9 28^ As discussed in earlier sections, the concept of persistence applies to a single behaviour towards a goal, but crucially also to the extended sequences of behaviours required to attain most rewarding outcomes relevant to humans. The ACC in particular has been implicated in maintaining these behaviours over extended time periods.^26^ Given the clear association between ACC disruption and apathy, and the association of altered mesolimbic dopaminergic systems with apathy in PD, failure of persistence towards a goal is another possible component that could contribute to the apathetic phenotype, and which future research could gainfully explore (s12).

However, it is not enough to blindly persist towards an earlier selected goal. Adaptive behaviour requires online monitoring of the chosen option against other possibilities—a process captured by foreground/background decision making and foraging models of behaviour.^42^ In these models, the crucial question is whether the value of the current behaviour (*What am I doing right now?*) is higher than the background average value of behaviours in the current environment (*What else could I be doing?*) (s13). An important question for future study is whether this class of decisions is altered in apathetic patients, and in particular whether the reward insensitivity observed in apathetic patients might lead to a chronic underestimation of the environmental reward context (*What else could I be doing?*), such that it is never worth switching activities (even if the current activity is simply sitting on the couch). Interestingly, such an alteration of behaviour has been reported in patients with schizophrenia, associated with their degree of anhedonia which, on the questionnaire used, likely reflected deficits in reward-seeking behaviour.^43^

#### Vigour

The vigour of response is another key component of instrumental behaviour.^28^ Importantly, motor responses towards rewarding targets were preserved in apathetic patients with PD in two separate studies, despite altered sensitivity to the rewarding outcome itself.^34 38^ These findings contrast with another investigation, which reported a reduction in motor vigour associated with apathy.^44^ Further studies specifically examining this issue will be important.

#### Learning

ACC and VS activity encodes information about the *outcomes* of our actions, and these signals can drive learning about which behaviours are worth performing in the future.^22 45^ How could this be relevant to apathy? Apathetic behaviour might potentially arise from systematic biases in learning about actions and their outcomes. A down-weighting of learning about how actions relate to rewarding outcomes could lead to reward insensitivity, while increased weighting between a course of action and its effort costs might result in hypersensitivity to these costs during subsequent decisions. Any change in the balance between these two learning processes could alter the weighing up of costs and rewards as an individual decides if an action *is worth it* (figure 3A). Notably, one group has reported such a change in outcome-related electrophysiological activity (associated with reward information) in apathetic patients with PD^33^ (figure 3E). Furthermore, a number of behavioural and imaging studies have demonstrated an association between impaired learning about rewards and amotivation in schizophrenia.^37^ These changes could conceivably result, over time, in the pattern of decision-making behaviour recently observed in apathetic patients with PD, in which a higher threshold of reward was required to trigger acceptance of effortful offers.^38^

### Other potential contributors to amotivated behaviour

There are also other cognitive processes relevant to goal-directed behaviour and thus potentially apathy. Furthermore, it is notable that changes in EBDM associated with apathy have tended to correlate with particular apathy domains—subscales that emphasise a person’s daily activities and ability to initiate these activities.^34 38^ The lack of correlation between these task metrics and other subscales (eg, those mapping onto cognitive or emotional dimensions of apathy) suggests there are other facets of what we currently conceptualise as apathy that may not be explained by deficits in EBDM alone. Nevertheless, the role in particular of emotional blunting in apathy, and how this relates to aspects of goal-directed behaviour such as reward valuation, is an important topic for further research. A recent synthesis of decision making and emotions proposed that emotions are simply states elicited by rewards and punishers, as the result of actions, and it is these states themselves that may form the goals of actions.^46^ This suggests that overlapping neural processes may underlie reward valuation and emotions, processes which depend crucially on structures within the medial frontal cortex.^14 46^ We next consider other phases of behaviour that may be relevant to the apathetic syndrome.

#### Option generation

The process of generating potential options for behaviour provides crucial inputs for subsequent decision making and has been linked to function within the anterolateral prefrontal cortex.^47^ Recent work has found that apathetic traits in healthy participants are associated with reduced option generation, although this same result was not seen in clinically apathetic patients with PD.^48^

#### Enacting behaviour towards goals

The ability to coordinate thoughts and actions in relation to internal goals is a multifaceted construct often subsumed within the term *cognitive control*.^49^ Key components include working memory, cognitive flexibility, inhibition and planning, often referred to collectively as *executive functions*. Most conceptualisations of apathy include a specific ‘cognitive’ component referring to dysfunction of the above processes, putatively associated with dorsolateral prefrontal cortex (DLPFC)-dorsal striatum loop damage.^2 50^ There is also some neuroimaging evidence associating DLPFC and apathy.^51^ It could be argued that such cognitive deficits reduce goal-directed behaviour in a way more akin to physical impairments preventing movements rather than being a primary deficit in motivation. This is because it is the inability to enact behaviour towards a goal rather than reduced drive towards the goal which is altered. Although there is some evidence for executive dysfunction associated with apathy, it is inconsistent between studies and patient groups^52^ (s14). This could relate to variation in the apathy syndrome, tasks lacking the fine-grained assessment required to detect more subtle changes, or confounding factors such as concurrent damage to other brain regions important for motivated behaviour. Further research is required to determine the specific impact of dysfunction of executive processes on motivated behaviour.

#### A role for bad habits?

That apathy is a disorder of goal-directed behaviour is essentially sine qua non.^2 53^ However, there is no empirical evidence to suggest other systems of behaviour are preserved in apathy, in particular habitual behaviour (figure 1). Many of the behaviours that apathetic patients do not do, such as cleaning the house or making a cup of tea, are probably normally performed under the guidance of habitual systems.^54^ Future research might investigate the formation and maintenance of habitual behaviour in apathetic patients. Demonstration of impairment within this system would have implications for both non-pharmacological (eg, overtraining patients on basic activities) and pharmacological treatments.

#### Environmental context

While the discussion thus far has focused on how disruption of normal cognitive processes could lead to apathy, it is also important to consider the context within which behaviour occurs. Simplistically, a person’s environment consists of reinforcers (both positive and negative) which can vary in terms of both quantity and predictability (s15). Positive reinforcers can be complex phenomenon such as conversing with a friend, being smiled at, successfully baking a cake, hearing a song on a radio and so on. A context in which there are few positive reinforcers, or in which it is difficult to learn the relationship between actions and positive outcomes, could also lead to, or exacerbate, apathetic behaviour. This is a particularly important point to consider because the diseases in which apathy occurs (table 1) also tend to predispose patients to be in such poorly reinforcing environments.

### Apathy and neuromodulatory systems—DA and beyond

There is a clear scientific basis for considering the mesolimbic dopaminergic system as a primary influence of and potential therapeutic target for apathy.^9 28 55^ However there is now evidence that DA exerts at least some of its actions on motivated behaviour along a dissociable axis to apathy.^38^ Furthermore, although some therapeutic trials have demonstrated improvement in apathy with dopaminergic manipulations,^56 57^ others have not.^58^

The neuroscience literature has begun to emphasise the importance of non-dopaminergic systems in aspects of motivated behaviour and EBDM.^11 20^ Preliminary clinical data have demonstrated the efficacy of agomelatine, a 5-HT2C receptor inverse agonist, in treating apathy in patients with frontotemporal dementia,^59^ while manipulations of the cholinergic system have also shown promise in PD.^60^ Interestingly, the effects of these neuromodulators on EBDM are still tied to the mesolimbic dopaminergic system.^19^ This illustrates the complexity inherent in understanding the role of specific neurotransmitters in goal-directed behaviour, particularly in the setting of neurodegenerative disorders that are characterised by loss of multiple neuromodulatory pathways. Nevertheless, as our understanding of these systems grows, the opportunity to tailor pharmacological therapies more closely to their desired therapeutic effect should arise.

## Concluding remarks

Conceptualising apathy in terms of our knowledge of the underlying basic neuroscience of motivated behaviour results in a framework that, to some extent, challenges the current nosological approach to apathy. The conceptual approach to apathy we suggest here is in line with a broader shift to understand neuropsychiatric syndromes in terms of underlying normal neurobiology. However, this approach remains limited by, on one side, an evolving (rather than settled) understanding of how motivated behaviour is produced, and on the other side only scanty empirical evidence for what cognitive processes are actually disrupted in patients with apathy. As we outline above, further research is required—at behavioural and physiological levels—to delineate the components of goal-directed behaviour that are impaired in apathy, whether these changes are general or disease-specific, and how they relate to changes in neuromodulatory systems. This research should ultimately aim, through a deeper understanding of the mechanisms disrupting motivated behaviour, to develop effective treatments for this debilitating syndrome.

Additional references can be found in the online supplementary material.
