# Supplementary material for: Brain mechanisms underlying apathy
Source: J Neurol Neurosurg Psychiatry. 2018 Oct 26;90(3):302–12. doi: 10.1136/jnnp-2018-318265 (PMC6518466; doi:10.1136/jnnp-2018-318265)
Supplement: Supplementary data [file jnnp-2018-318265supp001.pdf]

For a diagnosis of Apathy the patient should fulfil the criteria A, B, C and D

A Loss of or diminished motivation in comparison to the patient's previous level of functioning and which is not consistent with his age or culture. These changes in motivation may be reported by the patient himself or by the observations of others.

B Presence of at least one symptom in at least two of the three following domains for a period of at least four weeks and present most of the time

Domain B1 : Loss of, or diminished, goal-directed behaviour as evidenced by at least one of the following:

- Loss of self-initiated behaviour (for example: starting conversation, doing basic tasks of day-to-day living, seeking social activities, communicating choices)
- Loss of environment-stimulated behaviour (for example: responding to conversation, participating in social activities)

Domain B2 : Loss of, or diminished, goal-directed cognitive activity as evidenced by at least one of the following:

- Loss of spontaneous ideas and curiosity for routine and new events (i.e., challenging tasks, recent news, social opportunities, personal/family and social affairs).
- Loss of environment-stimulated ideas and curiosity for routine and new events (i.e., in the persons residence, neighbourhood or community)

Domain B3 : Loss of, or diminished, emotion as evidenced by at least one of the following:

- Loss of spontaneous emotion, observed or self-reported (for example, subjective feeling of weak or absent emotions, or observation by others of a blunted affect)
- Loss of emotional responsiveness to positive or negative stimuli or events (for example, observer-reports of unchanging affect, or of little emotional reaction to exciting events, personal loss, serious illness, emotional-laden news)

C These symptoms (A—B) cause clinically significant impairment in personal, social, occupational, or other important areas of functioning.

D The symptoms (A—B) are not exclusively explained or due to physical disabilities (e.g. blindness and loss of hearing), to motor disabilities, to diminished level of consciousness or to the direct physiological effects of a substance (e.g. drug of abuse, a medication).

Task Force agreed criteria to diagnose apathy – from *Robert et al 2009*, with permission.

Robert P, Onyike CU, Leentjens AFG, Dujardin K, Aalten P, Starkstein S, et al. Proposed diagnostic criteria for apathy in Alzheimer's disease and other neuropsychiatric disorders. Eur Psychiatry. 2009;24(2):98–104.

Animal models of motivational dysfunction

Recent research has focused upon the development of formal animal models of effort-related motivational dysfunction, which could contribute to the development of treatments for apathy, anergia, and fatigue symptoms seen across multiple disorders. Much recent research has focused on the use of tetrabenazine (TBZ) for inducing impairments in effort-related decision making (1). TBZ reversibly inhibits the type-2 vesicular monoamine transporter, which blocks vesicular storage and results in a depletion of monoamines, with particularly strong effects on DA at low doses. Recent studies have shown that the effort-related impairments induced by TBZ across multiple tasks can be reversed by adenosine A2A antagonists (1,2) and also by DAT inhibitors (1,2) but not by drugs that block SERT or NET (3). Similar results have been observed in studies inducing effort-related dysfunction by administering pro-inflammatory cytokines (4).

1. Nunes EJ, Randall PA, Podurgiel S, Correa M, Salamone JD. Nucleus accumbens neurotransmission and effort-related choice behavior in food motivation: effects of

drugs acting on dopamine, adenosine, and muscarinic acetylcholine receptors. Neurosci Biobehav Rev. 2013 Nov;37(9 Pt A):2015–25.

2. Yohn SE, Santerre JL, Nunes EJ, Kozak R, Podurgiel SJ, Correa M, et al. The role of dopamine D1 receptor transmission in effort-related choice behavior: Effects of D1 agonists. Pharmacol Biochem Behav. 2015 Aug;135:217–26.
3. Yohn SE, Collins SL, Contreras-Mora HM, Errante EL, Rowland MA, Correa M, et al. Not All Antidepressants Are Created Equal: Differential Effects of Monoamine Uptake Inhibitors on Effort-Related Choice Behavior. Neuropsychopharmacology. 2016 Feb;41(3):686–94.
4. Salamone JD, Yohn SE, López-Cruz L, San Miguel N, Correa M. Activational and effort-related aspects of motivation: neural mechanisms and implications for psychopathology. Brain. 2016 May;139(5):1325–47.
