# Supplementary material for: Brain mechanisms underlying apathy
Source: J Neurol Neurosurg Psychiatry. 2018 Oct 26;90(3):302–12. doi: 10.1136/jnnp-2018-318265 (PMC6518466; doi:10.1136/jnnp-2018-318265)
Supplement: Supplementary data [file jnnp-2018-318265supp002.pdf]

Supplementary Online References

s1. Locke J. An Essay Concerning Human Understanding, by John Locke : B2.7. 1689 Essay 2.7.3. Available from: https://ebooks.adelaide.edu.au/l/locke/john/l81u/B2.7.html
s2. Thompson JC, Harris J, Sollom AC, Stopford CL, Howard E, Snowden JS, et al. Longitudinal evaluation of neuropsychiatric symptoms in Huntington's disease. J Neuropsychiatry Clin Neurosci. 2012;24(1):53–60. Available from: http://www.ncbi.nlm.nih.gov/pubmed/22450614
s3. Niv Y, Daw ND, Joel D, Dayan P. Tonic dopamine: opportunity costs and the control of response vigor. Psychopharmacology (Berl). 2007 Apr;191(3):507–20.
s4. Silvetti M, Alexander W, Verguts T, Brown JW. From conflict management to reward-based decision making: Actors and critics in primate medial frontal cortex. Neurosci Biobehav Rev. 2014;46(P1):44–57. Available from: http://dx.doi.org/10.1016/j.neubiorev.2013.11.003
s5. Sutton RS, Barto AG. Reinforcement learning : an introduction. MIT Press; 1998. 322 p. Available from: https://mitpress.mit.edu/books/reinforcement-learning
s6. Otto AR, Gershman SJ, Markman AB, Daw ND. The Curse of Planning. Psychol Sci. 2013 May 4;24(5):751–61. Available from: http://www.ncbi.nlm.nih.gov/pubmed/23558545
s7. Randall PA, Pardo M, Nunes EJ, López Cruz L, Vemuri VK, Makriyannis A, et al. Dopaminergic Modulation of Effort-Related Choice Behavior as Assessed by a Progressive Ratio Chow Feeding Choice Task: Pharmacological Studies and the Role of Individual Differences. Beeler JA, editor. PLoS One. 2012 Oct 22;7(10):e47934. Available from: http://dx.plos.org/10.1371/journal.pone.0047934
s8. Cousins MS, Sokolowski JD, Salamone JD. Different effects of nucleus accumbens and ventrolateral striatal dopamine depletions on instrumental response selection in the rat. Pharmacol Biochem Behav. 1993 Dec 1;46(4):943–51. Available from: https://www.sciencedirect.com/science/article/pii/009130579390226J
s9. Yohn SE, Santerre JL, Nunes EJ, Kozak R, Podurgiel SJ, Correa M, et al. The role of dopamine D1 receptor transmission in effort-related choice behavior: Effects of D1 agonists. Pharmacol Biochem Behav. 2015 Aug;135:217–26. Available from: http://linkinghub.elsevier.com/retrieve/pii/S0091305715001446
s10. Floresco SB, Ghods-Sharifi S. Amygdala-Prefrontal Cortical Circuitry Regulates Effort-Based Decision Making. Cereb Cortex. 2006 Feb 22;17(2):251–60. Available from: http://www.ncbi.nlm.nih.gov/pubmed/16495432
s11. Manohar SG, Husain M. Reduced pupillary reward sensitivity in Parkinson's disease. npj Park Dis. 2015 Dec 17;1(1):15026. Available from: http://www.nature.com/articles/npjparkd201526
s12. Holroyd CB, Umemoto A. The research domain criteria framework: The case for anterior cingulate cortex. Neurosci Biobehav Rev. 2016 Dec;71:418–43. Available from: http://linkinghub.elsevier.com/retrieve/pii/S0149763416303645
s13. Stephens DW, Krebs JR (John R. Foraging theory. Princeton University Press; 1986. Available from: http://press.princeton.edu/titles/2453.html
s14. Baggio HC, Segura B, Garrido-Millan JL, Marti MJ, Compta Y, Valldeoriola F, et al. Resting-state frontostriatal functional connectivity in Parkinson's disease-related apathy. Mov Disord. 2015 Apr 15;30(5):671–9. Available from: http://doi.wiley.com/10.1002/mds.26137
s15. Behrens TEJ, Woolrich MW, Walton ME, Rushworth MFS. Learning the value of information in an uncertain world. Nat Neurosci. 2007 Sep 5;10(9):1214–21. Available

from: http://www.nature.com/articles/nn1954
s16. Lanctôt KL, Agüera-Ortiz L, Brodaty H, Francis PT, Geda YE, Ismail Z, et al. Apathy associated with neurocognitive disorders: Recent progress and future directions. Alzheimer's Dement. 2017 Jan;13(1):84–100. Available from: http://www.ncbi.nlm.nih.gov/pubmed/27362291
s17. Ligthart SA, Richard E, Fransen NL, Eurelings LSM, Beem L, Eikelenboom P, et al. Association of vascular factors with apathy in community-dwelling elderly individuals. Arch Gen Psychiatry. 2012;69(6):636–42. Available from: http://www.ncbi.nlm.nih.gov/pubmed/22664551
s18. Hollocks MJ, Lawrence AJ, Brookes RL, Barrick TR, Morris RG, Husain M, et al. Differential relationships between apathy and depression with white matter microstructural changes and functional outcomes. Brain. 2015 Dec;138(12):3803–15. Available from: http://www.ncbi.nlm.nih.gov/pubmed/26490330
s19. Reyes S, Viswanathan A, Godin O, Dufouil C, Benisty S, Hernandez Z, et al. Apathy: A major symptom in Cadasil. Neurology. 2009;72(10):905–10.
s20. Caeiro L, Ferro JM, Costa J. Apathy secondary to stroke: A systematic review and meta-analysis. Cerebrovasc Dis. 2013;35(1):23–39.
s21. Lansdall CJ, Coyle-Gilchrist ITS, Jones PS, Vázquez Rodríguez P, Wilcox A, Wehmann E, et al. Apathy and impulsivity in frontotemporal lobar degeneration syndromes. Brain. 2017 Jun;140(6):1792–807. Available from: http://www.ncbi.nlm.nih.gov/pubmed/28486594
s22. Litvan I, Mega MS, Cummings JL, Fairbanks L. Neuropsychiatric aspects of progressive supranuclear palsy. Neurology. 1996 Nov;47(5):1184–9. Available from: http://www.ncbi.nlm.nih.gov/pubmed/8909427
s23. Litvan I, Cummings JL, Mega M. Neuropsychiatric features of corticobasal degeneration. J Neurol Neurosurg Psychiatry. 1998 Nov;65(5):717–21. Available from: http://www.ncbi.nlm.nih.gov/pubmed/9810944
s24. Lillo P, Mioshi E, Zoing MC, Kiernan MC, Hodges JR. How common are behavioural changes in amyotrophic lateral sclerosis? Amyotroph Lateral Scler. 2011 Jan 19;12(1):45–51. Available from: http://www.tandfonline.com/doi/full/10.3109/17482968.2010.520718
s25. Arnould A, Rochat L, Azouvi P, Van der Linden M. A Multidimensional Approach to Apathy after Traumatic Brain Injury. Neuropsychol Rev. 2013 Sep 7;23(3):210–33. Available from: http://www.ncbi.nlm.nih.gov/pubmed/23921453
s26. Hoare J, Fouche J-P, Spottiswoode B, Joska JA, Schoeman R, Stein DJ, et al. White matter correlates of apathy in HIV-positive subjects: a diffusion tensor imaging study. J Neuropsychiatry Clin Neurosci. 2010 Aug 1;22(3):313–20. Available from: http://neuro.psychiatryonline.org/cgi/doi/10.1176/appi.neuropsych.22.3.313
s27. Paul RH, Brickman AM, Navia B, Hinkin C, Malloy PF, Jefferson AL, et al. Apathy is Associated With Volume of the Nucleus Accumbens in Patients Infected With HIV. J Neuropsychiatry Clin Neurosci. 2005 May;17(2):167–71. Available from: http://psychiatryonline.org/doi/abs/10.1176/jnp.17.2.167
s28. Rosti-Otajärvi E, Hämäläinen P. Behavioural symptoms and impairments in multiple sclerosis: a systematic review and meta-analysis. Mult Scler J. 2013 Jan;19(1):31–45. Available from: http://www.ncbi.nlm.nih.gov/pubmed/22383231
s29. Rubinsztein JS, Rubinsztein DC, Goodburn S, Holland AJ. Apathy and hypersomnia are common features of myotonic dystrophy. J Neurol Neurosurg Psychiatry 1998 Apr;64(4):510–5. Available from: http://www.ncbi.nlm.nih.gov/pubmed/9576545
s30. Svetel M, Potrebić A, Pekmezović T, Tomić A, Kresojević N, Jesić R, et al. Neuropsychiatric aspects of treated Wilson's disease. Parkinsonism Relat Disord. 2009

Dec;15(10):772–5. Available from: http://linkinghub.elsevier.com/retrieve/pii/S1353802009001448
s31. Treadway MT, Zald DH. Reconsidering anhedonia in depression: lessons from translational neuroscience. Neurosci Biobehav Rev. 2011 Jan;35(3):537–55. Available from: http://linkinghub.elsevier.com/retrieve/pii/S0149763410001120
s32. Foussias G, Remington G. Negative symptoms in schizophrenia: avolition and Occam's razor. Schizophr Bull. 2010 Mar;36(2):359–69. Available from: http://www.ncbi.nlm.nih.gov/pubmed/18644851
s33. Aberman JE & Salamone JD. Nucleus accumbens dopamine depletions make rats more sensitive to high ratio requirements but do not impair primary food reinforcement. Neuroscience. 1999;92(2):545-52.
s34. Robert G, Le Jeune F, Lozachmeur C et al. Preoperative factors of apathy in subthalamic stimulated Parkinson disease. A PET study. Neurology. 2014 83:1620-26.
s35. Schroeter M, Vogt B, Frisch S et al. Dissociated behavioral disorders in early dementia – An FDG-PET study. Psychiatry Research: Neuroimaging. 2011 194: 235-244.
